# On-Chip Immunoelectrophoresis of Extracellular Vesicles Released from Human Breast Cancer Cells

**DOI:** 10.1371/journal.pone.0123603

**Published:** 2015-04-30

**Authors:** Takanori Akagi, Kei Kato, Masashi Kobayashi, Nobuyoshi Kosaka, Takahiro Ochiya, Takanori Ichiki

**Affiliations:** 1 Department of Bioengineering, School of Engineering, The University of Tokyo, 2-11-16 Yayoi, Bunkyo-ku, Tokyo, Japan; 2 Division of Molecular and Cellular Medicine, National Cancer Center Research Institute, 5-1-1 Tsukiji, Chuo-ku, Tokyo Japan; Istituto Superiore di Sanita, ITALY

## Abstract

Extracellular vesicles (EVs) including exosomes and microvesicles have attracted considerable attention in the fields of cell biology and medicine. For a better understanding of EVs and further exploration of their applications, the development of analytical methods for biological nanovesicles has been required. In particular, considering the heterogeneity of EVs, methods capable of measuring individual vesicles are desired. Here, we report that on-chip immunoelectrophoresis can provide a useful method for the differential protein expression profiling of individual EVs. Electrophoresis experiments were performed on EVs collected from the culture supernatant of MDA-MB-231 human breast cancer cells using a measurement platform comprising a microcapillary electrophoresis chip and a laser dark-field microimaging system. The zeta potential distribution of EVs that reacted with an anti-human CD63 (exosome and microvesicle marker) antibody showed a marked positive shift as compared with that for the normal immunoglobulin G (IgG) isotype control. Thus, on-chip immunoelectrophoresis could sensitively detect the over-expression of CD63 glycoproteins on EVs. Moreover, to explore the applicability of on-chip immunoelectrophoresis to cancer diagnosis, EVs collected from the blood of a mouse tumor model were analyzed by this method. By comparing the zeta potential distributions of EVs after their immunochemical reaction with normal IgG, and the anti-human CD63 and anti-human CD44 (cancer stem cell marker) antibodies, EVs of tumor origin circulating in blood were differentially detected in the real sample. The result indicates that the present method is potentially applicable to liquid biopsy, a promising approach to the low-invasive diagnosis of cancer.

## Introduction

Extracellular vesicles (EVs) are small lipid vesicles ranging in diameter from 30 to 1,000 nm that are released from cells and exist stably in body fluids such as blood, saliva, urine, and cerebrospinal fluid [1]. EV is a generic term for secreted vesicles including exosomes, microvesicles, and apoptotic bodies [2]. Among them, exosomes are relatively small vesicles ranging in diameter from 30 to 100 nm that are of endocytic origin with a specific composition including proteins, lipids, and nucleic acids [3,4]. Over the last decade, intensive studies on exosome biology have revealed many important findings. Exosomes are recognized as mediators of intercellular communication in the immune system, cancer development, and cancer metastasis by transferring functional molecules such as protein, mRNA, and microRNA (miRNA) from one cell to another; consequently, they regulate gene expression in recipient cells at the post-transcriptional level [5–12]. In addition to the academic interest and importance in fundamental biology, exosomes are attracting much attention because they are considered to be useful for therapeutic and diagnostic applications [13–16]. Exosomes are reported to be promising biomarker candidates for the diagnosis of various diseases including cancer [4,16–18], renal diseases [19], inflammation [20], and metabolic disorders [21].

Although the biological characteristics and functions of exosomes are becoming increasingly clear, many researchers have pointed out that conventional analytical methods are not sufficient for characterizing nanoparticles such as exosomes [22–26]. Because the samples collected from living bodies are essentially a heterogeneous assortment of diverse types with vesicles of different cell origins and biogenetic mechanisms, methods that can characterize individual small particles ranging in diameter from 30 to 400 nm are desired.

A powerful approach to characterizing heterogeneous biological samples is immunoassay method using antibodies for specific molecular recognition. Particle/cell immunoelectrophoresis is one such immunoassay method that can provide qualitative and quantitative information on surface molecules of particles including cells and organelles. In our previous study, we developed a method of on-chip cell immunoelectrophoresis that allows rapid and accurate characterization of the immunogenicity of individual cells [27]. The antibody binding on the cell surface was detected on a microcapillary electrophoresis (μCE) chip by measuring changes in the electrophoretic velocity of cells by particle tracking velocimetry. In analogy, the extended use of on-chip immunoelectrophoresis is expected to be a promising method for the surface marker analysis of individual EVs, but it requires some technical problems to be overcome such as the implementation of an appropriate imaging system that enables the tracking of the electrophoretic migration of individual vesicles smaller than the wavelength of light [28,29].

In this paper, we present the first report on the on-chip immunoelectrophoresis of EVs for the simple-to-use and robust surface marker profiling of individual EVs. Immunoelectrophoresis experiments were successfully performed on EV samples collected from the culture supernatant of human breast cancer cells using an anti-human CD63 antibody, which is enriched in exosomes and microvesicles [30,31]. Moreover, we attempted the specific detection of EVs of tumor origin by the on-chip immunoelectrophoresis of EVs collected from the blood of mouse tumor models and discuss its applicability to liquid biopsy, the low-invasive diagnosis of cancer, and other diseases.

## Materials and Methods

### Cell culture

MDA-MB-231 human breast cancer cells were cultured in Roswell Park Memorial Institute (RPMI) medium (GIBCO) supplemented with 10% fetal bovine serum (FBS), 100 μg/ml kanamycin, 100 units/ml penicillin, and 100 μg/ml streptomycin in a humidified atmosphere of 95% air and 5% CO_2_ at 37°C.

### Mouse study

All experimental procedures and animal handling were performed according to the guidelines of National Cancer Center Research Institute, Tokyo, Japan. The protocol was approved by the committee on the Ethics of Animal Experiments of the National Cancer Center (Permit Number: T12-011). Four-week-old female BALB/c athymic nude mice (CLEA Japan) were anesthetized by 3% isoflurane injections. MDA-MB-231 cells (2×10^6^ cells in 100 μl of phosphate-buffered saline [PBS]) were injected into the subcutaneous tissue of anesthetized mice. Blood samples of approximately 1 ml were taken by heart puncture at the time of sacrifice of each mouse on the 40th day after injection with the cancer cells. All surgery was performed under isoflurane anesthesia, and all efforts were made to minimize suffering. The animals were used for only one measurement in each experiment.

### Collection of EV samples

EVs released from cultivated MDA-MB-231 cells were collected in accordance with the procedure described previously [32]. The culture medium was replaced with a serum-free RPMI medium and the supernatant was collected after incubation for 48 h. EVs were collected from the supernatant by differential ultracentrifugation performed as follows. The samples were centrifuged twice at 2,000×*g* for 15 min using a centrifuge (Tomy LC-220) and the resulting supernatant was recentrifuged at 12,000×*g* for 35 min using an ultracentrifuge (Beckman Coulter Optima L-90K). Subsequently, the clarified supernatant was ultracentrifuged at 110,000×*g* for 70 min. The resulting pellets were washed in 30 ml of PBS (1.06 mM KH_2_PO_4_, 155.17 mM NaCl, 2.97 mM Na_2_HPO_4_·7H_2_O, pH = 7.4, ionic strength = 0.16, GIBCO) and finally centrifuged at 110,000×*g* for 70 min. The washed pellets containing EVs were then suspended in PBS.

Similarly, EV samples were isolated from the mouse tumor model. Pooled plasma samples were obtained by the centrifugation of blood of 10 mice at 3,000 rpm for 1 min at 4°C. The pooled plasma sample was necessary to perform the on-chip immunoelectrophoresis because of the low plasma volume of one mouse. Consequently, the detection of individual differences was avoided as same as previous EV studies [33–35]. Then, EVs were collected from the plasma by differential ultracentrifugation by the procedure described above.

### Size distribution measurement

The size distribution of the collected EVs was evaluated using a nanoparticle tracking analysis (NTA) system (Nanosight system, Malvern) [36]. The Brownian motion of each EV was visualized by a light scattering method and recorded for 30 s, and then, specifically tracked to calculate its size using the Stokes—Einstein equation.

### On-chip immunoelectrophoresis

The zeta potential of EVs was measured using an electrophoresis platform developed in our laboratory [12,28,37]. Briefly, the platform comprises a μCE chip, a pair of platinum electrodes, a DC power supply [Matsusada Precision HVL-1.1P(A)], a 488 nm laser source (Melles Griot 85-BCD-030-053, 50 mW), a microscope (Nikon, Ti-U), and an electron-multiplying charge-coupled device (EM-CCD) camera (Andor iXon3 897). Poly(dimethylsiloxane) (PDMS)-based μCE chips were fabricated by the soft lithography method [38]. To suppress nonspecific adsorption and the generation of electroosmotic flow (EOF), the surface of a rectangular microchannel (length, 10,000 μm; width, 100 μm; height, 50 μm) was coated with a phospholipid copolymer containing 2-methacryloyloxyethyl phosphorylcholine (MPC) and 3-methacryloxyethyl triethoxysilane (METESi) [39,40].

EVs suspended in PBS were incubated with normal mouse IgG (isotype control), an anti-human CD63 (hCD63) mouse antibody or an anti-human CD44 (hCD44) mouse antibody for 30 min at 4°C. All the antibodies used in this study were purchased from BD Biosciences. The final antibody concentration was adjusted to 10 μg/ml. After dispensing a small amount of the suspension of the EV and antibody mixture (approximately 150 μl) on a μCE chip, the chip was placed on the stage of an inverted microscope, and then electrodes were dipped into the reservoirs at both ends of the microchannel. The electrophoresis experiment was performed by applying an electric field of 50 V/cm in the microchannel. The motion of individual EVs was visualized by laser dark-field imaging and their migration velocity was measured from the recorded video. Because the measured velocity of migrating EVs is affected by the EOF of the buffer solution in the microcapillary, the true electrophoretic velocity of EVs, *U*_ep_, was calculated by subtracting the EOF velocity, *U*_eo_, which was measured using charge-free beads, from the measured velocity of EVs, *U*_m_. The zeta potential of EVs, ζ, was calculated from the measured electrophoretic velocity using the Smoluchowski equation

$$\zeta =\frac{\varepsilon_{0}\varepsilon_{r}}{\eta }\cdot \frac{U_{\text{ep}}}{E}=\frac{\varepsilon_{0}\varepsilon_{r}}{\eta }\cdot \frac{U_{\text{m}}-U_{\text{eo}}}{E}\;,$$ (1)

where *E* is the electric field strength,*n* is the viscosity coefficient of the buffer solution, and ε_*r*_ and ε_*o*_ are the relative permittivity of the solution and the permittivity of vacuum, respectively.

### Statistical analysis

The statistical significance of differences between groups was evaluated by the Steel-Dwass test. A probability level of P<0.05 was considered to be significant.

## Results and Discussion

### Immunoelectrophoresis of EVs purified from cell culture supernatant

**Fig 1:**
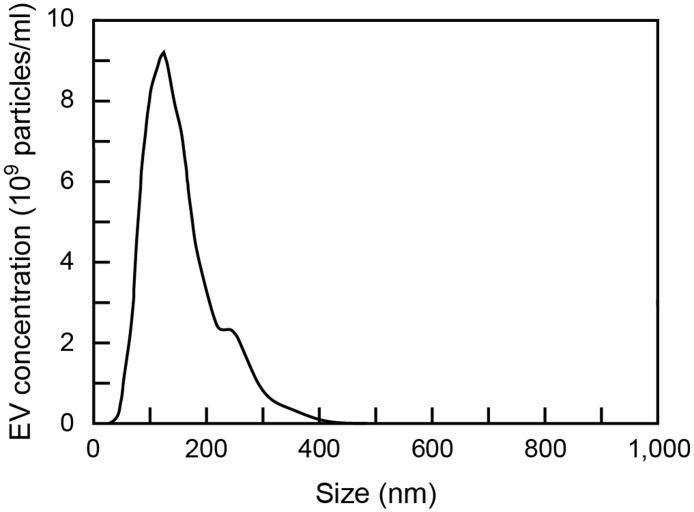
Size distribution of EVs collected from cell culture supernatant of MDA-MB-231 human breast cancer cells and evaluated using nanoparticle tracking analysis (NTA) system.

Prior to the zeta potential measurement, the concentration and size distribution of EVs were measured using an NTA system. Fig 1 shows the typical size distribution of EVs collected from the culture supernatant of MDA-MB-231 cells. The EV concentration of this sample was 1.19×10^12^ particles/ml. The broad size distribution ranging from approximately 50 nm to 450 nm implies that the collected sample contains different types of EV. Although vesicle types cannot be strictly classified by size [24], it is known that exosomes range between 30 and 100 nm in diameter [41] and that most microvesicles range between 100 and 300 nm [41,42]. Thus, the EV samples used in this study were considered to contain both exosomes and microvesicles, where the latter were in the majority. However, it is noted that the number of exosomes might be rather underestimated because NTA does not yet have the sensitivity to detect vesicles smaller than 50 nm.

**Fig 2:**
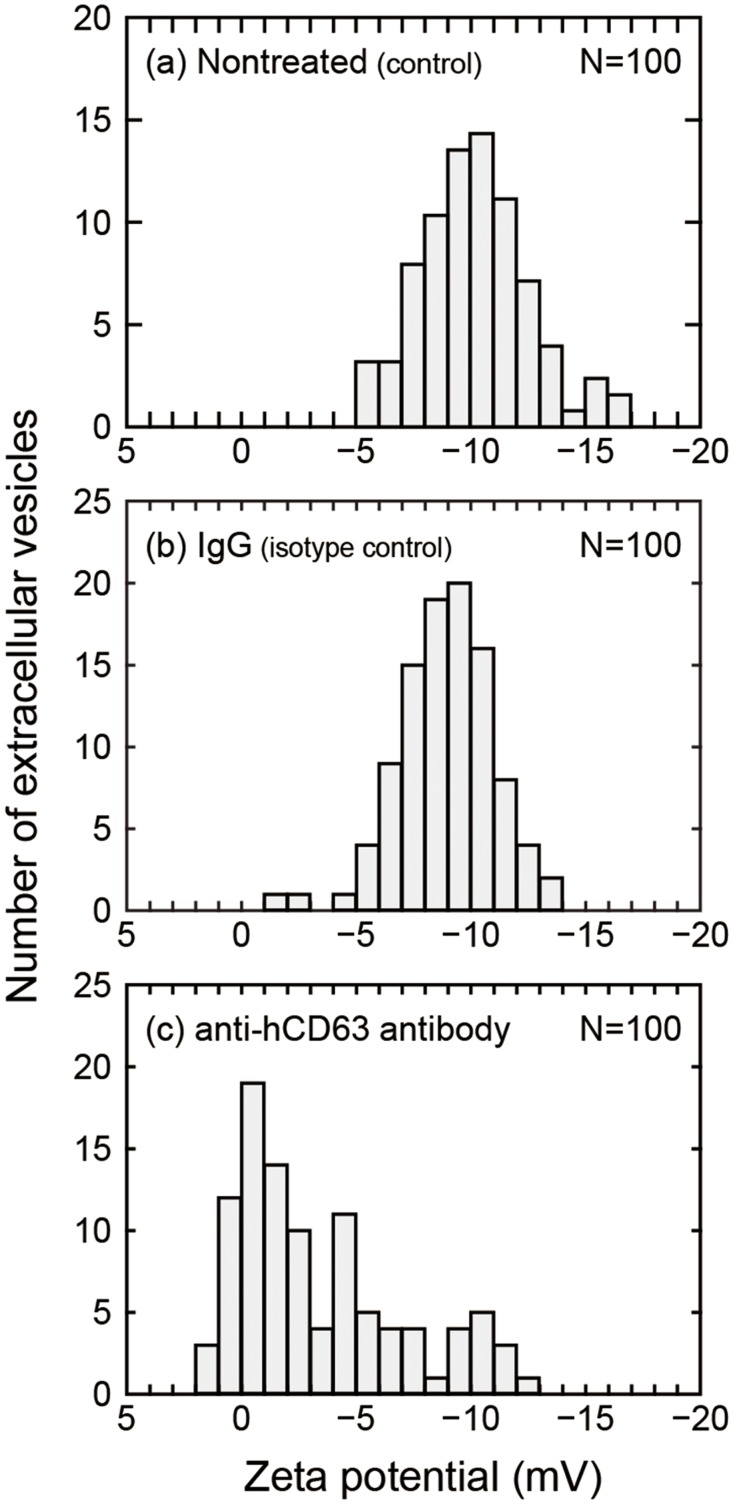
Zeta potential of EVs collected from culture medium of MDA-MB-231 human breast cancer cells and evaluated without any antibodies (a) and after treatment with normal mouse IgG (b) and anti-hCD63 antibody (c). The zeta potential of EVs with the anti-hCD63 antibody was statistically different from that without any antibodies and with normal-IgG (p<0.05, respectively, the Steel-Dwass test). CD63 is a marker protein of exosomes and microvesicles. The number of measured vesicles is 100 for each distribution.

**Fig 3:**
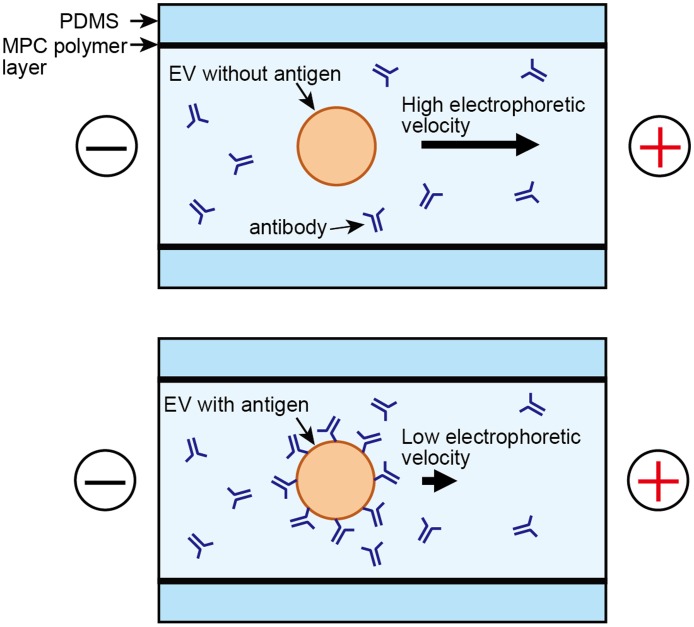
Schematic of immunoelectrophoresis of EVs on μCE chip. Because antibody binding increases the number of positive charges on the EV surface, the immunoreactivity of individual EVs is reflected in their electrophoretic mobility, and hence, their zeta potential. To suppress nonspecific adsorption and electroosmotic flow, the inner surface of the microchannel was coated with 2-methacryloyloxyethyl phosphorylcholine (MPC) polymer.

The on-chip immunoelectrophoresis of EVs was examined using the mouse anti-hCD63 antibody and normal mouse IgG. CD63 is a member of the tetraspanin family that constitutes the main component of the lysosomal membrane and is enriched on microvesicles as well as on exosomes [31]. For this reason, CD63 was used as a marker of exosomes and microvesicles in this paper. Normal IgG was used as an isotype control antibody to estimate the nonspecific binding of antibodies on the EV surface. As shown in Figs 2(a) and 2(b), the zeta potentials of EVs without any antibodies added and with normal IgG showed symmetric and unimodal distributions. The mean and median of the distribution were approximately equal at −10.2 mV and −10.1 mV for nontreated EVs and −9.0 mV and −9.1 mV for normal-IgG-treated EVs. Only a slight change was observed in the zeta potentials of EVs between the two groups. In contrast, as shown in Fig 2(c), a left-skewed distribution was observed with a peak near 0 mV and a mean and median of −3.4 mV and −2.1 mV, respectively, when EVs were immunoreacted with the anti-hCD63 antibody. The zeta potential of EVs with the anti-hCD63 antibody was statistically different from that without any antibodies and with normal-IgG (p<0.05, respectively, the Steel-Dwass test). As schematically depicted in Fig 3, the positive shift of the zeta potential observed for the anti-hCD63 antibody is attributed to the binding of positively charged antibodies on EVs that originally have negative charges. Here, it is worth commenting on the concentrations of the antibodies used in the present immunoelectrophoresis experiment. The concentration of the anti-hCD63 antibody was ~10^-7^ M, the total concentration of antigen proteins expressed on EVs was estimated to be <10^-7^, and the dissociation constant for the binding of CD63 (*K*_d_) was estimated to be ~10^-8^ M [43,44]. Hence, almost all of the CD63 molecules on EV surfaces are considered to bind to the antibody molecules. Thus, the shift of the zeta potential is expected to be proportional to the number density of the bound antibody molecules on the EV surface, and hence it can be used as a measure for quantifying the relative number density of marker proteins expressed on EVs.

### Immunoelectrophoresis of EVs collected from blood of tumor mouse model

**Fig 4:**
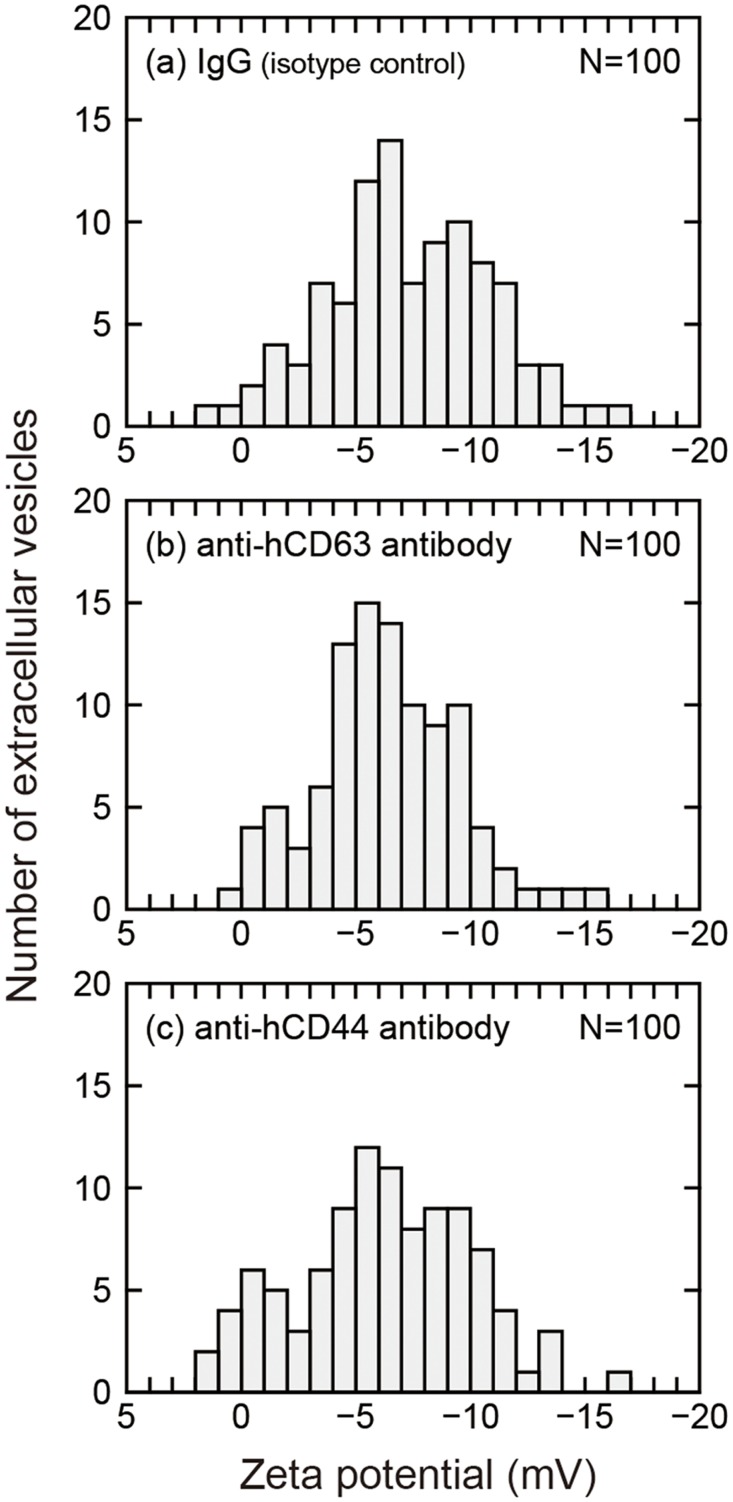
Zeta potential distribution of EVs collected from the pooled plasma sample of 10 orthotopic mouse models of breast cancer measured by on-chip immunoelectrophoresis using normal mouse IgG (a), mouse anti-human CD63 antibody (b), and mouse anti-human CD44 antibody (c). The zeta potentials of EVs with the anti-hCD63 antibody and with the anti-hCD44 antibody were statistically different from that with normal-IgG (p<0.05, respectively, the Steel-Dwass test). CD63 is a marker protein of exosomes and microvesicles. CD44 is used as a marker of MDA-MB-231 cell origin. Normal IgG was used as an isotype control to estimate the nonspecific binding of antibodies. The number of measured vesicles is 100 for each distribution.

As described in the above section, on-chip immunoelectrophoresis is a promising analytical method for characterizing the surfaces of individual EVs. Another concern about the present method is its applicability to disease diagnosis. Compared with the cell culture supernatant, blood samples contain a more heterogeneous population of EVs owing to the diverse cell type of origin, so both sensitivity and specificity are required. To explore the applicability of on-chip immunoelectrophoresis to cancer diagnosis by liquid biopsy using EVs as biomarkers, a proof-of-concept study was carried out using EVs collected from the pooled blood plasma of 10 orthotopic mouse models of breast cancer. In addition to the anti-hCD63 antibody and normal IgG used as described in the above section, the anti-hCD44 antibody was also used. CD44 is a transmembrane adhesion glycoprotein, functioning as a hyaluronan receptor and participating in the metabolism of its principal ligand hyaluronan [45], and it is abundant in MDA-MB-231 cells [46]. Fig 4 shows the zeta potential distributions of EVs collected from the blood of the mice and treated with the three different antibodies. The zeta potential of normal-IgG-treated EVs shows a broader distribution and rather more complicated histogram than that shown in Fig 2(b) of EVs released only from MDA-MB-231 cells. This finding seems to reflect the diversity of cell origin and biogenetic mechanisms in the animal body. By comparing the zeta potential distributions of EVs after immunoreactions with the normal IgG, anti-hCD63 antibody, and anti-hCD44 antibody, the zeta potentials of EVs with the anti-hCD63 antibody and with the anti-hCD44 antibody were statistically different from that with normal-IgG (p<0.05, the Steel-Dwass test). Furthermore, we observed additional peaks near 0 mV for both the anti-hCD63 and anti-hCD44 antibodies. Note that these peak positions are in good agreement with those in Fig 2(c). Hence, the additional peaks are most reasonably attributed to the immunoreactions between the antibodies and EVs. In particular, a bimodal distribution is clear in the case of the anti-hCD44 antibody, as shown in Fig 4(c). Thus, EVs of human tumor origin circulating in blood were differentially detected in the real sample, which inevitably also contains EVs of mouse nontumor origin. In the future it is necessary to evaluate the difference between normal and tumor derived vesicles extracted from the plasma of healthy persons and cancer patients for application development. Although the present feasibility study was performed for cancer diagnosis, such an EV-based diagnosis is expected to be applicable to various diseases with the advances in the development of exosome biomarkers.

### Comparative discussion of surface marker analysis of individual EVs

**Fig 5:**
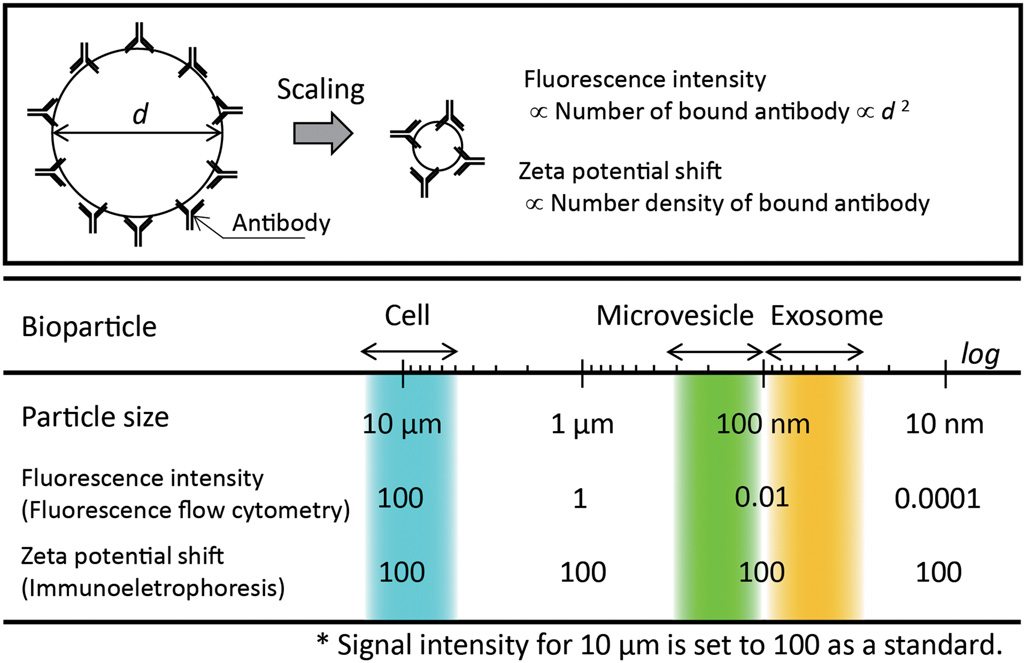
Comparison of size scaling of signal intensity in surface marker detection between fluorescence flow cytometry and on-chip immunoelectrophoresis. Schematic illustration of scaling effect on particle-antibody complexes (upper figure). Relationship between signal intensity and size of bioparticles (lower figure).

Hereafter, we discuss the unique characteristics of the present method as a tool for surface marker analysis in comparison with flow cytometry. Fluorescence flow cytometry is a golden standard for the surface marker analysis of cells and is also considered to be applicable to the characterization of individual EVs in principle. Practically, however, it is difficult to use conventional flow cytometry for analyzing EVs. The reason for this is obvious when considering the scaling of measured physical parameters to the diameter *d*, as illustrated in Fig 5. Assuming that the measured vesicles have the same chemical components, the fluorescence signal intensity decreases rapidly with decreasing *d* as the square of *d* because the number of attached fluorescently labeled antibodies is considered to be proportional to the surface area. Practically, the decrease in the intensity of the light scattering signals as the sixth power of *d* is also a serious problem in operating a fluorescence flow cytometer because it is usually used as a trigger signal for fluorescence measurement. Although recent improvements of high-resolution flow-cytometry-based methods have enabled the detection and analysis of fluorescently labeled EVs of ~100 nm [47], the requirement for an experienced operator and expensive apparatus will limit their widespread use. Additionally, the use of high-power lasers usually induces the problematic phenomenon of photobleaching.

In contrast, immunoelectrophoresis does not require fluorescent labeling for detecting bound antibodies; hence, photobleaching is no longer a problem. Additionally, the most important point is that the shift in the zeta potential is not scaled to the size of vesicles as long as the number density of bound antibody molecules is not changed. The main factor that determines the performance limit of on-chip immunoelectrophoresis is the sensitivity of the imaging system used for particle tracking analysis. Practically, the combined use of laser dark-field imaging and a sensitive EM-CCD camera enables the detection of particles with a diameter of 50 nm or smaller. Thus, on-chip immunoelectrophoresis is advantageous for the surface marker analysis of EVs including exosomes.

## Conclusion

To enable the simple-to-use and robust surface marker analysis of individual EVs, on-chip particle immunoelectrophoresis has been studied using a platform comprising a μCE chip and a laser dark-field imaging system. The immunoelectrophoresis of EVs collected from the culture supernatant of MDA-MB-231 human breast cancer cells was performed using mouse anti-hCD63 antibody, which is an exosome and microvesicle marker, and the result was compared with that for the normal mouse IgG isotype control. The zeta potential of EVs evaluated using normal IgG showed a symmetric and unimodal distribution with a peak near −9 mV, whereas a left-skewed distribution was observed with a peak near 0 mV when the anti-hCD63 antibody was used. Thus, immunogenicity of EVs was detected successfully as the positive shift of the zeta potential. Moreover, the zeta potential of EVs collected from the blood of nude mice implanted with MDA-MB-231 cells was evaluated by on-chip immunoelectrophoresis using the anti-hCD44 and anti-hCD63 antibodies. By analyzing the difference in the result between the isotype control and the antibodies, we observed additional peaks that indicate the formation of an antibody-EV complex near 0 mV for both the anti-hCD44 and anti-hCD63 antibodies. The approach described here is expected to facilitate the surface marker analysis of EVs circulating in blood as a noninvasive “liquid biopsy” for personalized medicine in the future. In particular, the sensitive profiling of EVs of tumor origin using a certain set of vesicle and tumor-specific antibodies will be useful for early cancer screening, tumor-type determination, prognosis, and the monitoring of treatment outcomes or tumor dynamics over time.
